# Matrix-M™ adjuvation broadens protection induced by seasonal trivalent virosomal influenza vaccine

**DOI:** 10.1186/s12985-015-0435-9

**Published:** 2015-12-08

**Authors:** Freek Cox, Eirikur Saeland, Matthijs Baart, Martin Koldijk, Jeroen Tolboom, Liesbeth Dekking, Wouter Koudstaal, Karin Lövgren Bengtsson, Jaap Goudsmit, Katarina Radošević

**Affiliations:** Present Address: Infectious Diseases and Vaccines Therapeutic area, Janssen Research and Development, Pharmaceutical companies of Johnson and Johnson, Leiden, The Netherlands; Present Address: Janssen Prevention Center, Center of Excellence of Janssen Research & Development, Janssen Pharmaceutical companies of Johnson and Johnson, Leiden, The Netherlands; Present Address: Novavax AB, Uppsala, Sweden; Present Address: Sanofi, Global Biotherapeutics, Vitry-sur-Seine, France

**Keywords:** Matrix-M™, Protection, Seasonal influenza vaccine, Adjuvant, Cross-reactive HAI response, Mice

## Abstract

**Background:**

Influenza virus infections are responsible for significant morbidity worldwide and therefore it remains a high priority to develop more broadly protective vaccines. Adjuvation of current seasonal influenza vaccines has the potential to achieve this goal.

**Methods:**

To assess the immune potentiating properties of Matrix-M™, mice were immunized with virosomal trivalent seasonal vaccine adjuvated with Matrix-M™. Serum samples were isolated to determine the hemagglutination inhibiting (HAI) antibody titers against vaccine homologous and heterologous strains. Furthermore, we assess whether adjuvation with Matrix-M™ broadens the protective efficacy of the virosomal trivalent seasonal vaccine against vaccine homologous and heterologous influenza viruses.

**Results:**

Matrix-M™ adjuvation enhanced HAI antibody titers and protection against vaccine homologous strains. Interestingly, Matrix-M™ adjuvation also resulted in HAI antibody titers against heterologous influenza B strains, but not against the tested influenza A strains. Even though the protection against heterologous influenza A was induced by the adjuvated vaccine, in the absence of HAI titers the protection was accompanied by severe clinical scores and body weight loss. In contrast, in the presence of heterologous HAI titers full protection against the heterologous influenza B strain without any disease symptoms was obtained.

**Conclusion:**

The results of this study emphasize the promising potential of a Matrix-M™-adjuvated seasonal trivalent virosomal influenza vaccine. Adjuvation of trivalent virosomal vaccine does not only enhance homologous protection, but in addition induces protection against heterologous strains and thus provides overall more potent and broad protective immunity.

**Electronic supplementary material:**

The online version of this article (doi:10.1186/s12985-015-0435-9) contains supplementary material, which is available to authorized users.

## Background

Influenza virus infections cause significant morbidity and mortality, with 5 million severely ill and 250 –500.000 deaths annually, in particular among the elderly, the immunocompromised and people with chronic diseases. The estimated global attack rate of influenza virus is 5 – 10 % for adults and 20 – 30 % for children, which causes large health and economic burdens for the society [1]. Circulating seasonal influenza strains belong to A and B viruses. Influenza A viruses are classified on the basis of the antigenic properties of their hemagglutinin (HA) and neuraminidase (NA) glycoproteins. To date, 18 HA subtypes and 9 NA subtypes have been identified [2]. Influenza B viruses are classified in two lineages, B/Yamagata and B/Victoria [3]. While the host range of influenza B viruses is limited to humans and seals [4], influenza A viruses infect a broad range of hosts including humans, birds and pigs [5]. There is a constant threat of influenza A viruses crossing the species barrier and causing serious disease burden in humans [6], as was recently demonstrated by human cases of avian H7N9 in China [7].

Current trivalent seasonal influenza vaccines (TIV) are designed to elicit protective immunity against two specific influenza A strains (H1N1 and H3N2) and one B strain. The vaccines are mainly based on HA and primarily induce antibodies directed to the receptor binding site located on the globular head of the HA molecule which prevent the interaction of the virus with host cells and thereby block viral entry. However, since the globular head of the HA is highly variable [8, 9] seasonal vaccines require annual updating to be effective. Each year the World Health Organization provides recommendations for the composition of seasonal influenza vaccines, based on predictions of the strains that will become dominant in the upcoming season. These predictions are based on global monitoring of the circulating H1N1, H3N2 and B strains, but this procedure is an error-prone process and mismatches between circulating virus and vaccine strains occur frequently. A particular challenge is posed by the fact that two B-strains (one from each lineage) are co-circulating. In the last decade, the dominant strain was correctly predicted in only 50 % of the cases [10, 11].

Despite influenza strain variability, the HA contains conserved epitopes that may be targeted by vaccination [12–15]. For the development of a broadly protective influenza vaccine that protects against mismatched seasonal strains and potential pandemic strains it may be beneficial to redirect the immune response towards such conserved epitopes.

A possible approach to this may be the use of adjuvants. It has been shown previously that adjuvants, such as MF59, the AS03 adjuvant system and saponin-based adjuvants have an ability to enhance and broaden the immune response elicited by vaccination [16–26].

Here we evaluated the ability of a seasonal trivalent virosomal vaccine (TVV) adjuvated with the saponin-based adjuvant Matrix-M™ [27, 28] to elicit heterologous hemagglutination inhibiting (HAI) antibodies and protection in mice. HAI antibodies are very potent at preventing the entry of influenza virus into the cell, through blocking the interaction between the HA head and cellular sialic acid receptors, and in this way fully prevent infection [29, 30]. Furthermore, the HAI titer is the only accepted correlate of protection for influenza vaccines [31].

We demonstrate that Matrix-M™-adjuvated TVV improves homologous HAI titers and protection. In addition, Matrix-M™-adjuvated TVV elicits heterologous HAI titers against influenza B, but not against influenza A, and provides protection against heterologous influenza strains in mice.

## Results

### Matrix-M™-adjuvated TVV elicits high homologous HAI and protection

To investigate whether Matrix-M™ can enhance the overall humoral immune responses after immunization with TVV, we first evaluated vaccine homologous HAI responses in mice against all three vaccine strains: H1N1, H3N2 and B.

**Fig. 1:**
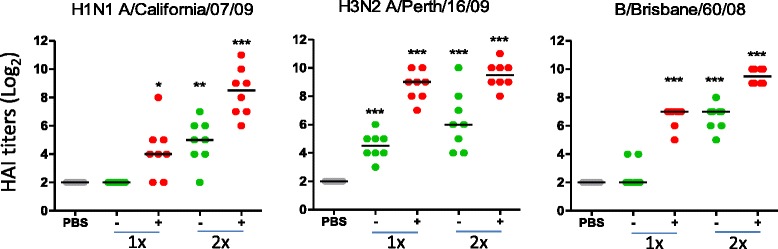
Matrix-M™ enhances homologous HAI responses induced by TVV. Mice (*n* = 8/group) were immunized with TVV (−) containing 3μg HA per strain or TVV + MM (+). Three weeks later, serum samples were obtained and tested for HAI responses against vaccine homologous H1N1 A/California/07/09, H3N2 A/Perth/16/09 (A/Victoria/210/09-like), and B/Brisbane/60/08 (B/Victoria lineage). Black bars indicate medians of log_2_ transformed titers. (*p < 0.05, **p < 0.01, ***p < 0.001, compared to the vehicle control group (PBS) using Wilcoxon’s rank-sum test adjusted for multiple comparisons). Results of the statistical comparisons between one and two immunizations and between immunizations with TVV and TVV + MM are summarized in Additional file 1: Table S1

A single immunization with a trivalent virosomal vaccine (TVV) alone did not result in HAI responses against the vaccine homologous H1N1 A/California/07/09 (non-significant compared to PBS, *p* = 1). Adjuvation with Matrix-M™ significantly enhanced the responses (*p* = 0.014 compared to 1xTVV), to a level comparable to a prime-boost immunization with TVV alone. A second immunization with Matrix-M™-adjuvated TVV (TVV + MM) further significantly improved the HAI titers (*p* = 0.003 compared to 1xTVV + MM, Fig. 1, left panel, Additional file 1: Table S1).

Mice immunized with a single dose of TVV alone had significant higher HAI titers against H3N2 A/Perth/16/09 (a strain that shows 98.8 % amino acid homology with the HA of the vaccine strain A/Victoria/210/09, Additional file 5: Figure S4) compared to PBS (*p* < 0.001) and adjuvation with Matrix-M™ further improved these responses (*p* < 0.001 compared to 1xTVV). Prime-boost immunization with TVV or TVV + MM did not further significantly enhance the HAI response as compared to single immunizations (*p* = 0.143 and *p* = 0.448, respectively, Fig. 1, middle panel).

A pattern similar to that of the HAI response against vaccine homologous H1N1 was observed for HAI responses against the B strain, where one immunization with TVV + MM induced HAI titers against the vaccine homologous B/Brisbane/60/08 strain (*p* < 0.001 compared to PBS), and up to the level comparable to prime-boost immunization with TVV alone. A second immunization with TVV + MM significantly boosted the primary HAI responses (*p* < 0.001 compared to 1xTVV + MM) (Fig. 1, right panel).

**Fig. 2:**
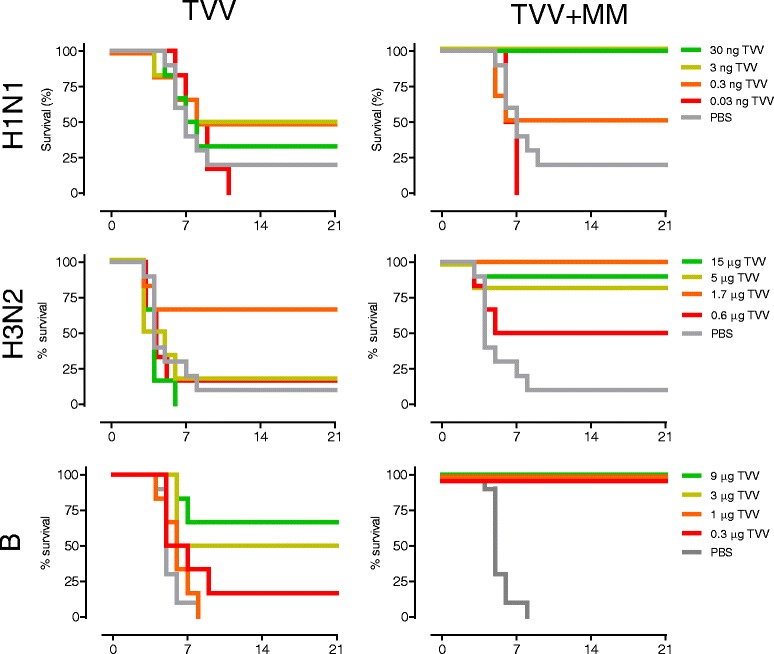
Survival proportion of homologous challenged mice. Mice (6 or 10/group) were immunized once with different doses of seasonal trivalent virosomal influenza vaccine (TVV) with or without Matrix-M™ or PBS and challenged with vaccine-homologous virus strains H1N1 A/Netherlands/602/09, H3N1 A/Perth/16/09 or B/Malaysia/2506/04 and monitored for 21 days for survival, body weight loss and clinical symptoms (Additional file 2: Figure S1). Graphs represent Kaplan-Meier survival curves. The HA amino acid sequences of H1N1 A/Netherlands/602/09 and H1N1 A/California/07/09 differ by 5 residues (99.5 % homology), those of H3N2 A/Perth/16/09 and H3N2 A/Victoria/210/09 differ by 7 residues (98.8 % homology), and those of B/Malaysia/2506/04 and B/Brisbane/60/08 differ by 5 residues (99.2 % homology) (Additional file 4: Figure S3, Additional file 5: Figure S4, Additional file 6: Figure S5)

Next we determined whether the enhanced homologous HAI titers obtained after Matrix-M™ adjuvation of TVV were associated with enhanced homologous protection. Therefore, mice were immunized with various suboptimal protective vaccine doses (suboptimal TVV doses were determined in a separate experiment, data not shown) with or without the addition of Matrix-M™ followed by intranasal challenge with vaccine homologous strains H1N1 A/Netherlands/602/09, H3N2 A/Perth/16/09 or B/Malaysia/2506/04 (B/Victoria lineage) (sequence alignments in Additional file 4: Figure S3, Additional file 5: Figure S4, Additional file 6: Figure S5). The results demonstrate that adjuvation with Matrix-M™ significantly improved TVV-induced protection against all three vaccine homologous strains as compared to TVV alone. (*p* = 0.026, *p* < 0.001 and *P* < 0.001 in an across doses comparison for the homologous H1N1, H3N2 and B challenges respectively) (Fig. 2 and Additional file 2: Figure S1).

### Matrix-M™-adjuvated TVV induces HAI responses against heterologous influenza B, but not against influenza A strains

**Fig. 3:**
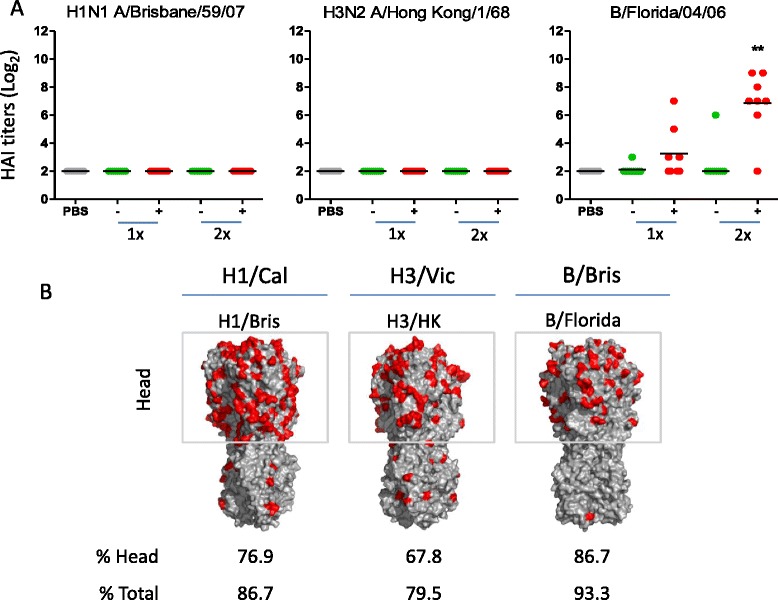
The addition of Matrix-M™ induces HAI responses against a heterologous influenza B strain but not against heterologous H1N1 and H3N2 strains. **a** Mice (*n* = 7-8/group) were immunized with TVV (−) containing 3μg HA per strain or TVV + MM (+). Three weeks later, serum samples were obtained and tested for HAI responses against H1N1 A/Brisbane/59/07 and H3N2 A/Hong Kong/1/68 and B/Florida/04/06 (B/Yamagata lineage). Black bars indicate medians of log_2_ transformed titers (*p < 0.05, **p < 0.01, ***p < 0.001 compared to the vehicle control group (PBS) using Wilcoxon’s rank-sum test adjusted for multiple comparisons). Comparisons between one and two immunizations and between immunizations with TVV and TVV + MM are summarized in Additional file 1: Table S1. **b** Numbers indicate HA homology as percentages of amino acid similarity (difference indicated in red) of the HA head region and of the whole HA sequence between vaccine strains and the assay virus strains (alignment of full length HA’s in Additional file 4: Figure S3, Additional file 5: Figure S4, Additional file 6: Figure S5). H1/Cal = A/California/07/09. H1/Bris = A/Brisbane/59/07. H3/Vic = A/Victoria/210/09. H3/HK = A/Hong Kong/1/68. B/Bris = B/Brisbane/60/08. B/Florida = B/Florida/04/06

In order to determine the breadth of the response, we analyzed HAI responses induced by TVV and TVV + MM against the heterologous influenza strains H1N1 A/Brisbane/59/07, H3N2 A/Hong Kong/1/68 and B/Florida/04/06 (B/Yamagata lineage). To compellingly assess the breadth of the response of TVV + MM we selected strains that show the highest difference possible in HA homology compared to the corresponding subtypes in the vaccine (the homology between the vaccine and challenge strains is depicted in Fig. 3b and Additional file 4: Figure S3, Additional file 5: Figure S4, Additional file 6: Figure S5).

HAI titers against heterologous H1N1 and H3N2 strains were not detected after 1 or 2 immunizations with TVV or TVV + MM (Fig. 3a). Furthermore, no HAI responses were detected against B/Florida/04/06 after one or two immunizations with TVV. In contrast, after a single immunization with TVV + MM, HAI titers were detected in 4 out of 8 mice, although the overall group response was not statistically significant (*p* = 0.154 compared to PBS). The HAI response to the heterologous B strain was increased after a second immunization with TVV + MM (*p* = 0.003 compared to PBS) (Fig. 3a). We tested a second strain of the B/Yamagata (B/Massachussets/01/12) lineage and found also for this strain comparable levels of HAI responses (Additional file 3: Figure S2B). These results demonstrate that adjuvation of TVV leads to induction of cross-reactive HAI against the two tested heterologous B strains but not against the two heterologous A strains.

### Matrix-M™-adjuvated TVV elicits protection against heterologous influenza strains

**Fig. 4:**
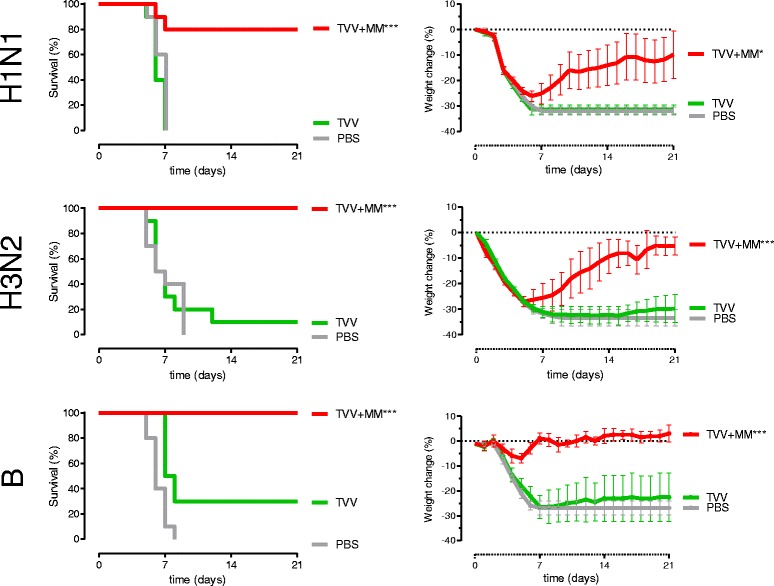
Matrix-M™ adjuvated TVV enhances protection against heterologous H1N1 H3N2 and influenza B strains. Mice (*n* = 10/group) were immunized once with TVV (containing 3μg HA per strain) with or without Matrix-M™. Four weeks later, mice were challenged with 25xLD_50_ of mouse-adapted heterologous H1N1 A/Brisbane/59/07, H3N2 A/Hong Kong/01/68 or B/Florida/04/06 and monitored for 21 days for survival, body weight loss (clinical symptoms, Additional file 3: Figure S2A). Graphs represent the Kaplan-Meier survival curves (left) and mean body weight change with 95 % confidence interval (right). Asterisks indicate statistically significant differences from the vehicle control group (*p < 0.05, ** *p* < 0.01, ***p < 0.001, according to the materials and methods section)

To investigate whether adjuvation of TVV with Matrix-M™ improves protection against heterologous strains, mice were immunized with a single dose of TVV or TVV + MM (corresponding to 3μg of each HA as was used in previous studies [32]). Four weeks later, mice were challenged with vaccine-heterologous wild-type derived mouse adapted H1N1, H3N2, or influenza B virus (see Fig. 3b, and sequence alignment Additional file 4: Figure S3, Additional file 5: Figure S4, Additional file 6: Figure S5), and monitored for 21 days for survival, body weight (Fig. 4) and clinical signs (Additional file 3: Figure S2A).

Despite the absence of detectable HAI titers against heterologous influenza A, Matrix-M™-adjuvated TVV was able to provide significant protection against mortality after heterologous challenge with H1N1 (A/Brisbane/59/07) and H3N2 (A/Hong Kong/1/68) strains while immunizations of non-adjuvated vaccine did not protect the animals against weight loss and clinical signs or mortality (Fig. 4). Although 80 % (H1N1) and 100 % (H3N2) of animals survived the challenge (*p* = 0.001 and *p* < 0.001 compared to PBS, respectively), they transiently lost substantial body weight and experienced severe clinical symptoms, indicating that this protection was not complete. However body weight loss (*p* = 0.015 and *p* = 0.001 respectively) and clinical scores (*p* < 0.001 and *p* < 0.001, respectively) were significantly lower compared to PBS.

We also investigated whether the enhanced cross-reactive HAI response translated into enhanced protection against vaccine heterologous B/Florida/04/06 (B/Yamagata lineage). A single immunization with TVV alone protected only 3 out of 10 mice, which was not statistically significant (*p* = 0.412 compared to PBS). The mice lost substantial body weight during the study period (*p* = 1 compared to PBS) and exhibited severe disease symptoms (*p* = 1 compared to PBS). In contrast, immunization with TVV + MM resulted in full protection (*p* < 0.001 compared to PBS) with hardly any body weight loss (*p* < 0.001 compared to PBS) and no clinical signs (*p* < 0.001 compared to PBS) after challenge with the heterologous influenza B strain (Fig. 4).

## Discussion

Influenza vaccines need to be annually updated to efficiently protect against circulating strains. Therefore, it remains a high priority to establish broad reactive immunity against influenza by vaccination. An attractive approach may be to attempt redirecting the immune response towards the conserved epitopes on the viral antigens by adjuvated seasonal influenza vaccines.

It has previously been demonstrated that adjuvants can enhance and broaden immune responses of seasonal and pandemic vaccines in animal models and in humans [16–24]. For example, MF59 (squalene oil-in-water emulsion [33] has been shown to enhance vaccine-elicited humoral immune responses in animals [20, 34, 35] and in humans [35–39] and it is currently licensed for use in combination with a seasonal influenza vaccine (Fluad®). Fluad and an MF59 adjuvated H5N1 pandemic vaccine candidate are known to induce H1 and H5 cross-reactive HAI responses in ferrets [20] and in humans [16, 17, 36, 39], respectively. In adults, cross-reactive HAI responses were also detected against drifted H3N2 influenza viruses [36]. MF59-adjuvated TIV also elicited some cross-reactive HAI responses against heterologous influenza B viruses in adults [37] but not in unprimed children [39].

Another adjuvant licensed for human use in combination with an H1N1 pandemic vaccine (Pandemrix®) is the AS03 adjuvant system (α-tocopherol and squalene in an oil-in-water emulsion [40]). It has been demonstrated, in pre-clinical and clinical studies, that the AS03-adjuvated H5N1 pandemic vaccines can elicit H5 cross-reactive HAI responses which correlated to protection [18, 19, 41].

In our studies, we have used Matrix-M™, an adjuvant that consists of a mixture of two purified and well characterized saponin fractions (Matrix-A and Matrix-C) [27]. Compared to the original formulation that contained non-fractionated Quillaja saponins together with viral antigens in a single particle [25], the individual particle formulation improves both adjuvant activity and the safety profile in humans [21]. Matrix-M™ in combination with TVV has been shown to enhance antibody and cellular immune responses against influenza in mice [22] and humans (Clinical-Trials.gov NCT01444482). Matrix-M™-adjuvated virosomal H9N2 and H5N1 vaccines have been evaluated in pre-clinical and clinical studies [21, 23, 42–45]. Mice immunized with an experimental H9N2 virosomal vaccine showed enhanced homologous HAI responses [45]. In addition, cross-clade H5 HAI responses were observed in mice that received a Matrix-M™-adjuvated H5N1 candidate vaccine compared to non-adjuvated H5N1 candidate pandemic vaccine in mice [23]. These findings were later confirmed in humans [21, 43].

Most studies concerning adjuvated influenza vaccines demonstrate cross-reactive responses against strains that are closely related to the vaccine strain.

Recently, we have shown that mice immunized twice with TVV + MM were protected from death after H5N1 and H7N7 challenge. Ferrets were partially protected against H5N1, but not against H7N9 after two immunizations with TVV + MM [26].

In our study the H1N1 and H3N2 challenge strains only show 87 % and 80 % homology (based on HA protein sequence) with the corresponding vaccine strains and yet a single immunization with TVV + MM could protect mice against these influenza A strains.

The HA among influenza B strains is more conserved compared to influenza A (>91 % based on HA protein sequence as determined by alignment of the circulating strains from 1970 to 2008, data not shown). Nonetheless, the current TIV formulations are inadequate to elicit protection against both influenza B lineages, particularly in naïve individuals [46, 47]. In addition, due to regular co-circulation of the strains from both lineages of influenza B, it is particularly difficult to predict which influenza B virus will be the dominant one in the upcoming season. The frequent mismatch between the influenza B lineage selected for seasonal vaccine and the dominant circulating B lineage results in the poor efficacy of seasonal vaccines against B strains [10]. One solution to improve vaccine efficacy against B strains is the inclusion of strains from both influenza B lineages in the vaccine, the so-called quadrivalent influenza vaccine (QIV). An alternative approach may be the enhancement and broadening of the vaccine-elicited immune response by an adjuvant such as Matrix-M™. Our results demonstrate that a Matrix-M™ adjuvated seasonal trivalent virosomal vaccine elicits HAI titers not only against vaccine homologous B/Brisbane/60/08 but also against the vaccine mismatched B/Florida/04/06 and B/Massachusetts/02/12 (93 % homology with the vaccine strain for both) In addition to the enhanced protection against the vaccine matched B strain, full protection against a lethal challenge with the B/Florida/04/06 strain was achieved.

In contrast to influenza B, Matrix-M™-adjuvated TVV did not elicit cross-reactive HAI responses against heterologous H1N1 or H3N2 influenza A strains. The lack of cross-reactive influenza A HAI response is in agreement with our previous study [26] and with studies that use a monovalent H1N1 split influenza vaccine formulated as ISCOMs [24, 48].

The presence of cross-reactive influenza B HAI and the absence of cross-reactive influenza A HAI can be explained by the higher homology between the HA head of the two B lineages, as compared to the homology for H1N1 and H3N2 strains (Fig. 3b and Additional file 2: Figure S1, Additional file 3: Figure S2, Additional file 4: Figure S3). Similarly, cross-reactive HAI responses to relatively more closely related H5N1 strains were shown to be induced in mice immunized with a Matrix-M™-adjuvated virosomal H5N1 vaccine (98.7 % and 96.5 % HA homology of the NIBRG-88 and IBCDCRG-6 strains compared to the NIBRG-14 vaccine strain, respectively) [23].

Considering that Matrix-M™-adjuvated TVV elicited HAI titers against two heterologous influenza B strains and provided full protection against challenge with the heterologous B/Florida/04/06, it is conceivable that HAI antibodies played a key role in protection, but does not exclude the contribution of other immunological mechanisms. Interestingly, despite the absence of HAI titers against the two heterologous influenza A strains, adjuvation with Matrix-M™ significantly improved TVV-induced protection against the heterologous H1N1 and H3N2 strains. In contrast to protection against heterologous influenza B, protection against influenza A was accompanied by the severe weight loss and disease symptoms. It is conceivable that the mechanism of heterologous protection induced by Matrix-M™-adjuvated TVV differs between influenza A and influenza B. We suggest that cross-reactive HAI antibodies play a key role in the protection against B/Florida/04/06. In the absence of cross-reactive HAI titers, as is the case for the two tested influenza A strains, other protective mechanisms are likely to play a key role. These include neutralization mediated by HA stem-binding antibodies [12–14], antibody dependent cellular cytotoxicity (ADCC) [24, 48–50], or T-cell mediated killing [24, 48, 51, 52]. The exact mechanism of protection against heterologous influenza A strains induced by Matrix-M™-adjuvated TVV remains to be identified.

## Conclusion

The results of this study together with our previous results [26] emphasize the promising potential of a Matrix-M™-adjuvated seasonal trivalent virosomal influenza vaccine. Adjuvation of trivalent virosomal vaccine does not only enhance homologous protection, but in addition induces protection against heterologous strains and thus provides overall more potent and broad protective immunity.

## Materials and methods

### Statement of ethics

All mouse and ferret experiments were performed in accordance with Dutch legislation on animal experiments and approved by the DEC Consult (Independent ethical institutional review board).

### Immunization

Six-to eight-week-old female BALB/c (H-2d) mice (specific pathogen-free) were purchased from Charles River (France). H1N1 A/California/07/09, H3N2 A/Victoria/210/09 and B/Brisbane/60/08 monovalent virosomes were prepared by Crucell (Berne, Switzerland) using conventional procedures [53]. The monovalent virosomes were mixed to obtain a trivalent virosomal vaccine (TVV). Matrix-M™ (MM, 10μg/dose, Novavax AB, Uppsala, Sweden) was mixed with TVV (at various doses, as indicated) before immunization (TVV + MM). Mice were immunized intramuscularly (i.m.) 1 to 3-times with TVV or TVV + MM 3 weeks apart with 100μl vaccine (50 μl per hind leg). Control groups received 100 μl PBS. Three weeks after the final immunization blood was collected to assess serum HAI responses (data shown for 1x and 2x immunization only). In the challenge experiments mice received mouse adapted influenza virus four weeks after the final immunization (data shown for 1x immunization only).

### Hemagglutination Inhibition (HAI) assay

HAI assays were performed as described before [45]. Briefly, sera were pre-absorbed with 0.5 % turkey red blood cells (bioTRADING Benelux B.V., Mijdrecht, the Netherlands) in PBS for 2 h. After removal by centrifugation, sera were treated for 16 h with receptor-destroying enzyme (Sigma-Aldrich; St. Louis, MO, USA; diluted 1:25 in PBS) The receptor-destroying enzyme was heat inactivated for 30 min at 56°C. Two-fold serial dilutions of the sera (initial dilution 1:8) were incubated for 1 h at room temperature with influenza virus (H1N1 A/California/07/09 reassortant (NYMC X-181), H1N1 A/Brisbane/59/07, H3N2 A/Perth/16/09, H3N2 A/Hong Kong/1/68, B/Brisbane/60/08 reassortant (NYMC BX-35), B/Florida/04/06, and B/Massachusetts/02/12) (see HA sequence alignment in Additional file 4: Figure S3, Additional file 5: Figure S4, Additional file 6: Figure S5) standardized to 8 hemagglutination units. Turkey red blood cells were added and incubated for 60 min before hemagglutination inhibition was determined. Each serum sample was tested in duplicate. Titers were expressed as the reciprocal of the highest dilution where complete agglutination inhibition was observed.

### Influenza challenge

Four weeks after the final immunization mice were anesthetized by intraperitoneal (i.p.) administration of 100 mg/kg ketamine (Nimatek® 100 mg/ml, Eurovet, Cuijk, the Netherlands) in combination with 20mg/kg xylazine (Sedamun® 20 mg/ml, Eurovet). Mice were challenged with 25xLD_50_ of (i) H1N1 A/Netherlands/602/09 or mouse-adapted H1N1 A/Brisbane/59/07 or B/Florida/04/06 (performed at Janssen Research and Development, Leiden, the Netherlands) or (ii) mouse-adapted H3N2 A/Perth/16/09, H3N2 A/Hong Kong/1/68 or B/Malaysia/2506/04 (performed at TNO Triskelion, Zeist, the Netherlands) (HA sequence alignment see Additional file 4: Figure S3, Additional file 5: Figure S4, Additional file 6: Figure S5) via the intranasal route (a total of 50μl, 25μl per nostril). Notably, all challenge strains were wild-type or wild-type derived and did not contain any PR8 derived segments.

After challenge, mice were monitored for weight-loss, clinical score and survival for 21 days or until humane endpoint. Clinical scores for challenges performed at Janssen Research and Development (i) were defined as: 0 = no clinical signs, 1 = rough coat, 2 = rough coat, less reactive, passive during handling, 3 = rough coat, rolled up, labored breathing, passive during handling, 4 = rough coat, rolled up, labored breathing, unresponsive (=humane endpoint) or found dead (=score 4).

Clinical scores for challenges at TNO (ii) were defined as 0 = no clinical signs, 1 = rough coat, 2 = rough coat, labored respiration 3 = rough coat, labored respiration, hunched posture and/or blepharospasm, 4 = rough coat, labored respiration, hunched posture, blepharospasm, lethargic and/or thin/dehydrated. Humane endpoint was defined as clinical score 4 for more than 2 consecutive days (=score 5) and found dead = score 5.

### Statistical analysis

Statistical differences between immunizations with TVV with or without Matrix-M™ relative to negative control group receiving PBS were evaluated for HAI titers. Additionally, the effect of two immunizations compared to one immunization and the effect of Matrix-M compared to TVV alone were determined (Additional file 1: Table S1). Data was log-transformed and comparisons between groups were made using the Wilcoxon rank-sum test with adjustment for multiple comparisons (2 fold Bonferroni and for comparisons with PBS a stepwise approach testing first 2x and then 1x vaccination).

In the challenge models the vaccine groups were compared to the vehicle control group for survival proportion, change in body weight and clinical scores. For survival proportion after challenge, a Fisher’s exact test was performed. For body weight loss and clinical score analysis, repeated measurements in the challenge phase were summarized as a single outcome per animal using an Area Under The Curve (AUC) approach where missing values for animals that died before day 21 were imputed with a last-observation-carried-forward method. Body weight data are expressed as the change relative to the day 0 measurement. The AUC was then defined as the summation of the area above and below the baseline. An ANOVA on AUC’s was done with group as explanatory factor. Clinical scores were summarized as AUC per mouse and groups were compared using a generalized linear model with a cumulative logit distribution to compare area under the curves for ordinal variable.

For the vaccine homologous challenges, survival proportion per dose was compared to the vehicle control group using a 2-sided Fisher’s exact test. Across doses TVV + MM was compared to TVV only in a logistic regression model with log(dose) as covariable, adjuvant as factor and their interaction.

Statistical analyses were performed using SAS version 9.2 (SAS Institute Inc. Cary, NC, USA) and SPSS version 20 (SPSS Inc., IL, USA). Statistical tests were conducted two-sided at an overall significance level of α = 0.05.

## Additional files

Additional file 1: Table S1. Summary statistical analysis HAI assays. (DOCX 17 kb) Additional file 2: Figure S1. Body weight loss and clinical scores following homologous challenges. Mice (6 or 10/group) were immunized once with different doses of seasonal trivalent virosomal influenza vaccine (TVV) with or without Matrix-M™ or PBS and challenged with vaccine-homologous virus strains H1N1 A/Netherlands/602/09, H3N1 A/Perth/16/09 or B/Malaysia/2506/04 and monitored for 21 days for survival, body weight loss and clinical symptoms. Graphs represent mean body weight changes with 95 % confidence interval and median clinical scores with interquartile range. The HA amino acid sequences of H1N1 A/Netherlands/602/09 and H1N1 A/California/07/09 differ by 5 residues (99.5 % homology), those of H3N2 A/Perth/16/09 and H3N2 A/Victoria/210/09 differ by 7 residues (98.8 % homology), and those of B/Malaysia/2506/04 and B/Brisbane/60/08 differ by 5 residues (99.2 % homology) (Additional file 4: Figure S3, Additional file 5: Figure S4, Additional file 6: Figure S5). (PDF 96 kb) Additional file 3: Figure S2. Clinical scores of heterologous challenges. A) Mice (*n* = 10/group) were immunized once with TVV (containing 3 μg HA per strain) with or without Matrix-M. Four weeks later, mice were challenged with 25xLD_50_ mouse-adapted heterologous H1N1 A/Brisbane/59/07, H3N2 A/Hong Kong/01/68 or B/Florida/04/06 and monitored for 21 days for survival, body weight loss and clinical symptoms Graphs represent median clinical scores with interquartile range. Asterisks indicate significance compared to the vehicle control group (**p* < 0.05, ** *p* < 0.01, ****p* < 0.001, according to the materials and methods section). B) Cross-reactive influenza B HAI titers induced upon TVV + MM immunization. Mice (*n* = 7-8/group) were immunized once or twice with TVV (−) (3μg HA per strain) or TVV + MM (+). Three weeks after the last immunization, serum samples were obtained and tested for HAI responses against B/Massachusetts/02/12 (B/Yamagata lineage). Black bars indicate medians of log_2_ transformed titers (**p* < 0.05, ***p* < 0.01, ****p* < 0.001 compared to the vehicle control group (PBS) using Wilcoxon’s rank-sum test adjusted for multiple comparisons). Comparisons between one and two immunizations and between immunizations with TVV and TVV + MM are summarized in Additional file 1: Table S1. (PDF 108 kb) Additional file 4: Figure S3. Alignment of H1N1 strains based on hemagglutinin amino acid sequence. H1/Cal = A/California/07/09 [GenBank: ACP44189.1], H1/NL = A/Netherlands/602/09 [GenBank :AGU92700], H1/Bris = A/Brisbane/59/07 [GenBank: ADE28750.1]. Blue bar indicates head domain defined as Cys_59_ to Cys_292_. (PDF 146 kb) Additional file 5: Figure S4. Alignment of H3N2 strains based on hemagglutinin amino acid sequence. H3/Vic = A/Victoria/210/09 [GenBank: AFM72883.1], H3/Perth = A/Perth/16/09 [GenBank: ACS71642.1], H3/HK = A/Hong Kong/1/68 [GenBank: ACU79871.1]. Blue bar indicated head domain defined as Cys_68_ to Cys_293_. (PDF 146 kb) Additional file 6: Figure S5. Alignment of B-strains based on hemagglutinin amino acid sequence. B/Bris = B/Brisbane/60/08 [GenBank: ACN29380.1], B/Mal = B/Malaysia/2506/04 [GenBank: ACR15732], B/Florida = B/Florida/04/06 [GenBank: ACF54246.1], B/Mass = B/Massachusetts/02/12 [GenBank: AGL06036.1]. Blue bar indicates head domain defined as Asn_58_ to Glu_307_. (PDF 156 kb)
