# Supplementary material for: Matrix-M™ adjuvation broadens protection induced by seasonal trivalent virosomal influenza vaccine
Source: Virol J. 2015 Dec 8;12:210. doi: 10.1186/s12985-015-0435-9 (PMC4672496; doi:10.1186/s12985-015-0435-9)
Supplement: Additional file 1: Table S1. — Summary statistical analysis HAI assays. (DOCX 17 kb) [file 12985_2015_435_MOESM1_ESM.docx]

**Supplementary table 1: Summary statistical analysis HAI assays**

|  | **Comparison of group** | 1x TVV | 1x TVV+MM | 2x TVV | 2x TVV+MM | 2x TVV | 2x TVV+MM | 1x TVV+MM | 2x TVV+MM |
| --- | --- | --- | --- | --- | --- | --- | --- | --- | --- |
| **Subtype/Lineage** | **with group** | PBS | PBS | PBS | PBS | 1x TVV | 1x TVV+MM | 1x TVV | 2x TVV |
| H1N1 | A/California/07/09 | 1 | 0.014 | 0.003 | <0.001 | 0.003 | 0.003 | 0.014 | 0.003 |
| H1N1 | A/Brisbane/59/07 | *nd* | *nd* | *nd* | *nd* | *nd* | *nd* | *nd* | *nd* |
| H3N2 | A/Perth/16/09 | <0.001 | <0.001 | <0.001 | <0.001 | 0.448 | 0.143 | <0.001 | 0.008 |
| H3N2 | A/Hong Kong/01/68 | *nd* | *nd* | *nd* | *nd* | *nd* | *nd* | *nd* | *nd* |
| B/Victoria | B/Brisbane/60/04 | 0.993 | <0.001 | <0.001 | <0.001 | <0.001 | <0.001 | <0.001 | <0.001 |
| B/Yamagata | B/Florida/04/06 | 1 | 0.154 | 1 | 0.003 | 0.022 | 1 | 0.359 | 0.005 |
| B/Yamagata | B/Massachusetts/02/12 | 1 | 0.933 | 0.400 | <0.001 | 0.945 | 0.001 | 0.480 | 0.001 |

Table summarizes *p*-values of various group comparisons of HAI data. Statistical analysis was performed as described in the material and methods section. TVV = Trivalent Virosomal Vaccine. MM = Matrix-M™. *nd* indicates that no statistical analyses was performed due to a lack of detectable HAI titers.
