# Supplementary figures and images for: Matrix-M™ adjuvation broadens protection induced by seasonal trivalent virosomal influenza vaccine
Source: Virol J. 2015 Dec 8;12:210. doi: 10.1186/s12985-015-0435-9 (PMC4672496; doi:10.1186/s12985-015-0435-9)

Supplementary Figure 1

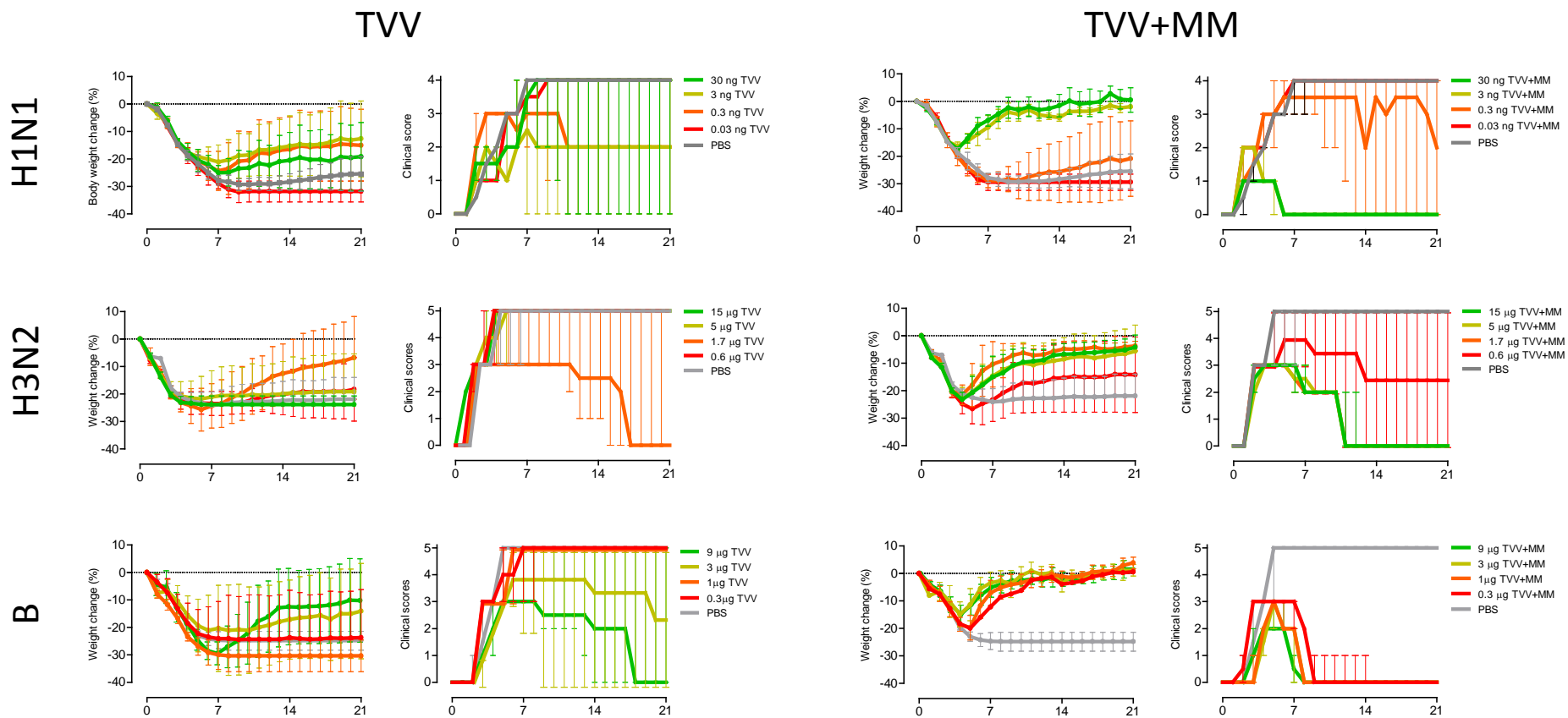

Supplement: Additional file 2: Figure S1. — Body weight loss and clinical scores following homologous challenges. Mice (6 or 10/group) were immunized once with different doses of seasonal trivalent virosomal influenza vaccine (TVV) with or without Matrix-M™ or PBS and challenged with vaccine-homologous virus strains H1N1 A/Netherlands/602/09, H3N1 A/Perth/16/09 or B/Malaysia/2506/04 and monitored for 21 days for survival, body weight loss and clinical symptoms. Graphs represent mean body weight changes with 95 % confidence interval and median clinical scores with interquartile range. The HA amino acid sequences of H1N1 A/Netherlands/602/09 and H1N1 A/California/07/09 differ by 5 residues (99.5 % homology), those of H3N2 A/Perth/16/09 and H3N2 A/Victoria/210/09 differ by 7 residues (98.8 % homology), and those of B/Malaysia/2506/04 and B/Brisbane/60/08 differ by 5 residues (99.2 % homology) (Additional file 4: Figure S3, Additional file 5: Figure S4, Additional file 6: Figure S5). (PDF 96 kb) [file 12985_2015_435_MOESM2_ESM.pdf]

Supplementary Figure 2

A

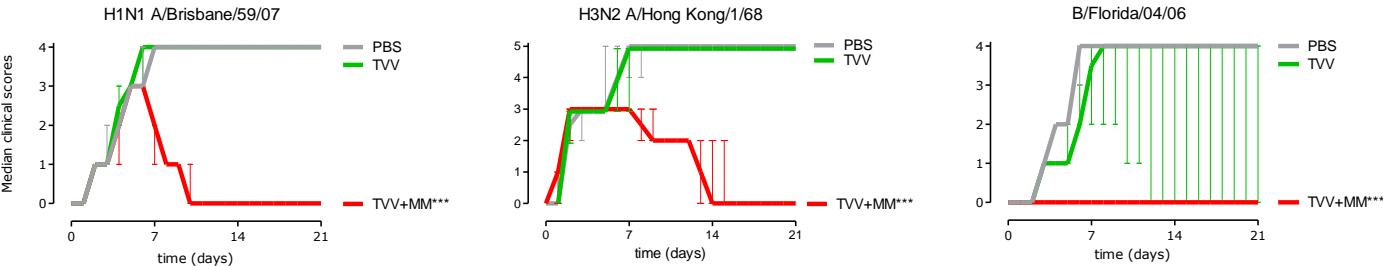

B

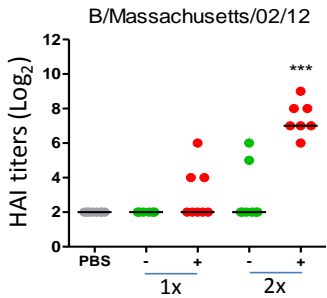

Supplement: Additional file 3: Figure S2. — Clinical scores of heterologous challenges. A) Mice (n = 10/group) were immunized once with TVV (containing 3 μg HA per strain) with or without Matrix-M. Four weeks later, mice were challenged with 25xLD50 mouse-adapted heterologous H1N1 A/Brisbane/59/07, H3N2 A/Hong Kong/01/68 or B/Florida/04/06 and monitored for 21 days for survival, body weight loss and clinical symptoms Graphs represent median clinical scores with interquartile range. Asterisks indicate significance compared to the vehicle control group (*p < 0.05, ** p < 0.01, ***p < 0.001, according to the materials and methods section). B) Cross-reactive influenza B HAI titers induced upon TVV + MM immunization. Mice (n = 7-8/group) were immunized once or twice with TVV (−) (3μg HA per strain) or TVV + MM (+). Three weeks after the last immunization, serum samples were obtained and tested for HAI responses against B/Massachusetts/02/12 (B/Yamagata lineage). Black bars indicate medians of log2 transformed titers (*p < 0.05, **p < 0.01, ***p < 0.001 compared to the vehicle control group (PBS) using Wilcoxon’s rank-sum test adjusted for multiple comparisons). Comparisons between one and two immunizations and between immunizations with TVV and TVV + MM are summarized in Additional file 1: Table S1. (PDF 108 kb) [file 12985_2015_435_MOESM3_ESM.pdf]
