# Supplementary material for: Matrix-M™ adjuvation broadens protection induced by seasonal trivalent virosomal influenza vaccine
Source: Virol J. 2015 Dec 8;12:210. doi: 10.1186/s12985-015-0435-9 (PMC4672496; doi:10.1186/s12985-015-0435-9)
Supplement: Additional file 5: Figure S4. — Alignment of H3N2 strains based on hemagglutinin amino acid sequence. H3/Vic = A/Victoria/210/09 [GenBank: AFM72883.1], H3/Perth = A/Perth/16/09 [GenBank: ACS71642.1], H3/HK = A/Hong Kong/1/68 [GenBank: ACU79871.1]. Blue bar indicated head domain defined as Cys68 to Cys293. (PDF 146 kb) [file 12985_2015_435_MOESM5_ESM.pdf]

Supplementary Figure 4

```
H3/Vic   MKTIIALSYILCLVFAQKLPGNDNSTATLCLGHHAVPNGTIVKTITNDQIEVTNATELVQNSSTGEICDSPHQILDGKNCTLIDA 85
H3/Perth ............................................................S........................ 85
H3/HK    ..........F..ALG.D......................L.....D.............S....K..NN..R....ID...... 85

H3/Vic   LLGDPQCDGFQNKKWDLFVERSKAYSNCYPYDVPDYASLRSLVASSGTLEFNNESFNWTGVTQNGTSSACIRRSKNSFFSRLNWL 170
H3/Perth ..................................................................................... 170
H3/HK    .....H..V...ET..........F..........................IT.G.T........G.N..K.GPGSG........ 170

H3/Vic   THLNFKYPALNVTMPNNEQFDKLYIWGVHHPVTDKDQIFLYAQASGRITVSTKRSQQTVIPNIGSRPRVRNIPTRISIYWTIVKP 255
H3/Perth ...............................G...........................S.............S........... 255
H3/HK    .KSGST..V........DN............S.NQE.TS..V.....V....R.....I........W..GLSS........... 255

H3/Vic   GDILLINSTGNLIAPRGYFKMQSGKSSIMRSDAPIGKCNSECITPNGSIPNDKPFQNVNRITYGACPRYVKQNTLKLATGMRNVP 340
H3/Perth ....................IR............................................................... 340
H3/HK    ..V.V...N............RT............DT.I....................K.......K................. 340

H3/Vic   EKQTRGIFGAIAGFIENGWEGMVDGWYGFRHQNSEGRGQAADLKSTQAAIDQINGKLNRLIGKTNEKFHQIEKEFSEVEGRIQDL 425
H3/Perth ..................................................................................... 425
H3/HK    ......L...............I.............T......................V.E....................... 425

H3/Vic   EKYVEDTKIDLWSYNAELLVALENQHTIDLTDSEMNKLFEKTKKQLRENAEDMGNGCFKIYHKCDNACIGSIRNGTYDHNVYRDE 510
H3/Perth ...............................................................................D..... 510
H3/HK    ..........................................RR.........................E.........D..... 510

H3/Vic   ALNNRFQIKGVELKSGYKDWILWISFAISCFLLCVALLGFIMWACQKGNIRCNICI 566
H3/Perth ........................................................ 566
H3/HK    ...................................V..........R......... 566
```
