# Supplementary material for: Matrix-M™ adjuvation broadens protection induced by seasonal trivalent virosomal influenza vaccine
Source: Virol J. 2015 Dec 8;12:210. doi: 10.1186/s12985-015-0435-9 (PMC4672496; doi:10.1186/s12985-015-0435-9)
Supplement: Additional file 6: Figure S5. — Alignment of B-strains based on hemagglutinin amino acid sequence. B/Bris = B/Brisbane/60/08 [GenBank: ACN29380.1], B/Mal = B/Malaysia/2506/04 [GenBank: ACR15732], B/Florida = B/Florida/04/06 [GenBank: ACF54246.1], B/Mass = B/Massachusetts/02/12 [GenBank: AGL06036.1]. Blue bar indicates head domain defined as Asn58 to Glu307. (PDF 156 kb) [file 12985_2015_435_MOESM6_ESM.pdf]

Supplementary Figure 5

```
B/Bris     MKAIIVLLMVVTSNADRICTGITSSNSPHVVKTATQGEVNVTGVIPLTTTPTKSHFANLKGTETRGKLCPKCLNCTDLDVALGRP 85
B/Mal      ..................................................................................... 85
B/Florida  ......................................................Y.......R.......D.............. 85
B/Mass     ......................................................Y.......K.......D.............. 85

B/Bris     KCTGKIPSARVSILHEVRPVTSGCFPIMHDRTKIRQLPNLLRGYEHIRLSTHNVINAENAPGGPYKIGTSGSCPNITNGNGFFAT 170
B/Mal      ....N......................................................................V......... 170
B/Florida  M.V.TT...KA......K...........................N.....Q...D..K......RL........A.SKS..... 170
B/Mass     M.V.TT...KA......K...................A.......N.....Q...D..K......RL........A.SKS..... 170

B/Bris     MAWAVPKNDKNKTATNPLTIEVPYICTEGEDQITVWGFHSDNETQMAKLYGDSKPQKFTSSANGVTTHYVSQIGGFPNQTEDGGL 255
B/Mal      .........N......S..........................I......................................... 255
B/Florida  .......-.N..N......V.....................DK...KN.....N....................S..D....... 254
B/Mass     .......-.N..N......V......A..............DK...KN.....N.......................D....... 254

B/Bris     PQSGRIVVDYMVQKSGKTGTITYQRGILLPQKVWCASGRSKVIKGSLPLIGEADCLHEKYGGLNKSKPYYTGEHAKAIGNCPIWV 340
B/Mal      ..................................................................................... 340
B/Florida  ...........M..P......V....V.......................................................... 339
B/Mass     ...........M..P......V....V.......................................................... 339

B/Bris     KTPLKLANGTKYRPPAKLLKERGFFGAIAGFLEGGWEGMIAGWHGYTSHGAHGVAVAADLKSTQEAINKITKNLNSLSELEVKNL 425
B/Mal      ..................................................................................... 425
B/Florida  ..................................................................................... 424
B/Mass     ..................................................................................... 424

B/Bris     QRLSGAMDELHNEILELDEKVDDLRADTISSQIELAVLLSNEGIINSEDEHLLALERKLKKMLGPSAVEIGNGCFETKHKCNQTC 510
B/Mal      ..................................................................................... 510
B/Florida  ..................................................................................... 509
B/Mass     ....................................................................D................ 509

B/Bris     LDRIAAGTFDAGEFSLPTFDSLNITAASLNDDGLDNHTILLYYSTAASSLAVTLMIAIFVVYMVSRDNVSCSICL 585
B/Mal      ........................................................................... 585
B/Florida  .........N.............................................L...I............... 584
B/Mass     .........N.............................................L...I............... 584
```
